# Regeneration of Cryoinjury Induced Necrotic Heart Lesions in Zebrafish Is Associated with Epicardial Activation and Cardiomyocyte Proliferation

**DOI:** 10.1371/journal.pone.0018503

**Published:** 2011-04-12

**Authors:** Kristin Schnabel, Chi-Chung Wu, Thomas Kurth, Gilbert Weidinger

**Affiliations:** Biotechnology Center and Center for Regenerative Therapies, Technische Universität Dresden, Dresden, Germany; National University of Singapore, Singapore

## Abstract

In mammals, myocardial cell death due to infarction results in scar formation and little regenerative response. In contrast, zebrafish have a high capacity to regenerate the heart after surgical resection of myocardial tissue. However, whether zebrafish can also regenerate lesions caused by cell death has not been tested. Here, we present a simple method for induction of necrotic lesions in the adult zebrafish heart based on cryoinjury. Despite widespread tissue death and loss of cardiomyocytes caused by these lesions, zebrafish display a robust regenerative response, which results in substantial clearing of the necrotic tissue and little scar formation. The cellular mechanisms underlying regeneration appear to be similar to those activated in response to ventricular resection. In particular, the epicardium activates a developmental gene program, proliferates and covers the lesion. Concomitantly, mature uninjured cardiomyocytes become proliferative and invade the lesion. Our injury model will be a useful tool to study the molecular mechanisms of natural heart regeneration in response to necrotic cell death.

## Introduction

All organisms have evolved means of dealing with tissue damage due to injury or disease. In most species, healing of epidermal wounds is an efficient process of repair, whereas the ability to recover from damage to other organs or structures varies widely. Mammals (including humans) can repair injury of skeletal muscle and peripheral nerves, and regenerate liver and pancreas, but have limited regenerative abilities with respect to other organs. In contrast, lower vertebrates, such as salamanders and fish, can completely regenerate lost limbs/fins and repair damage to lens, retina, kidney, and central nervous system [1], [2], [3]. Intriguingly, these organisms can also regenerate the heart [4], [5]. This stands in marked contrast to the mammalian heart, where tissue damage after ischemic cell death caused by myocardial infarction is irreversible because the mammalian heart is not capable of sufficient tissue regeneration [6], [7], [8]. Instead, the injured myocardium is replaced by a scar. This lack of tissue repair results in permanently reduced cardiac function after injury and will eventually lead to heart failure. To compensate for reduced cardiac function, the myocardium can undergo some hypertrophy, but does not produce new cardiomyocytes (CMs).

Intriguingly however, the adult mammalian heart contains resident progenitor cells that have the potential to differentiate into CMs (for review see [8]). These cells might play a role in homeostatic replacement of dying CMs, but they appear not to be activated for replacement of injured cells. Furthermore, attempts to achieve tissue replacement by transplantation of progenitor cells of various kinds into infarcted hearts have also generally failed (for review see [6], [7], [8]). The observed small improvement of heart function in such experiments is likely due to secretion of factors from the transplanted cells that positively influence vascularization and heart remodelling. Damaged myocardium is also not replaced from surviving CMs, since mature adult mammalian CMs are largely postmitotic and do not proliferate in response to injury. Interestingly, however, recent studies have shown that proliferation of mammalian cardiomyocytes can be re-activated by experimental modulation of signaling pathways [9]. These findings indicate that mammalian CMs are not principally unable to proliferate, and that it might be possible to activate intrinsic regenerative potential in the injured heart using the correct molecular signals.

In contrast to mammals, zebrafish, *Danio rerio*, efficiently repair damaged myocardium by production of new CMs in the absence of scar formation [4], [10]. Zebrafish are an excellent model to study the mechanisms of natural heart regeneration due to a wealth of accumulated knowledge about heart development and the availability of many molecular, genetic and genomic tools [11], [12], [13]. Furthermore, due to their short generation time, small size and cheap husbandry, a high number of individual fish can be studied and transgenic and mutant lines can be produced quickly and at relatively low cost. The zebrafish heart is also easily accessible for surgical or other experimental manipulations, the animals are highly tolerant to experimental cardiac injury and display robust and efficient heart regeneration after ventricular resection [10].

After surgical removal of up to 20% of the zebrafish ventricle the wound is sealed with a blood clot within minutes [4], [14]. A few days after amputation (dpa), profound transcriptional responses occur in the entire epicardium, including upregulation of genes normally expressed during development, which is followed by proliferation and thickening of the epicardial cell layer [15]. Subsequently, increased expression of developmental markers can be detected at the amputation plane, presumably in CMs [15]. CMs close to the amputation plane change their ultrastructure, sarcomeres disassemble and large dysmorphic mitochondria appear while total mitochondrial density is reduced [16], [17]. Therefore CMs have been proposed to de-differentiate in response to injury. In transgenic fish expressing the red fluorescent protein DsRed under control of the CM specific promoter *cardiac myosin light chain* (*cmlc2, myl7*), a population of CMs expressing low levels of DsRed starts to be detectable at around 7 dpa in the wound area [15], [16]. These presumably represent CMs that have only recently started to redifferentiate and reactivated the transgene and thus have not yet accumulated high levels of DsRed. At the same time, proliferation of both old and newly formed CMs can be detected [15]. Thus, zebrafish heart regeneration involves replacement of the resected myocardium with newly forming cardiomyocytes.

Recent genetic lineage tracing data using the Cre-Lox system has indicated that the entire regenerated myocardium is derived from existing, differentiated CMs [16], [17]. In particular, a subepicardial population of CMs that upregulate a *gata4* promoter fragment after ventricular resection appears to produce the bulk of the regenerated myocardial tissue [16]. Thus, a model emerges in which CMs in the subepicardial space dedifferentiate in response to injury, re-enter the cell cycle and proliferate to replace the missing tissue. At 60 dpa, most of the wound has been resolved, the missing myocardium has been replaced and no scar tissue has formed [4], [14]. Thus, zebrafish completely regenerate surgically removed myocardium.

Although the regenerative capacity of the zebrafish heart is impressive, it has remained untested whether it is associated with the type of injury that has so far been used, tissue removal. Clinically relevant models of heart injuries in mammals involve tissue death, typically due to ischemia [18]. It is conceivable that necrotic tissue represents an obstacle to regeneration that also the zebrafish cannot overcome. To address this, we have established a cryoinjury model of the adult zebrafish heart. We find that zebrafish robustly regenerate ventricular necrotic lesions and that the regenerative response involves early activation of the epicardium and induction of cardiomyocyte proliferation. Thus, our results show that the regenerative abilities of the zebrafish heart are not restricted to damage by tissue removal and that similar cellular mechanisms underlie regeneration after resection and cryoinjury. Our injury model will be of great use for studies of the molecular mechanisms of heart repair.

## Results and Discussion

### Zebrafish regenerate cryoinjury-induced lesions

**Figure 1:**
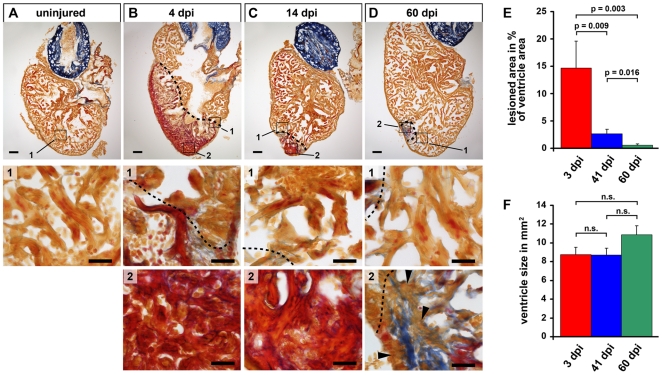
The zebrafish heart regenerates after cryoinjury induced tissue damage. (A–D) Sections of uninjured control hearts (A) and hearts at 4 (B), 14 (C) and 60 (D) days after cryolesion (dpi  =  days post injury) stained with Acid Fuchsin Orange G, which labels cardiomyocytes in orange, fibrin in red and collagen in blue. Magnification #1 of uninjured heart shows healthy myocardium. Magnifications #1 of injured hearts show cardiomyocytes at the lesion edges and parts of wound tissue. Close up views of the lesioned area are shown in magnifications #2. Note that the lesioned area, showing fibrin accumulation at 4 dpi and 14 dpi and minor deposits of collagen at 60 dpi is decreasing in size. Cardiomyocytes are missing at 4 and 14 dpi and at 60 dpi remaining collagen rich tissue is interspersed with cardiomyocytes (arrowheads). Wound edges are indicated by a black dashed line. Scale bars: 100 µm in overviews, 25 µm in close ups. (E) Quantification of the lesioned area normalized to the size of the ventricle. Measurements were performed on all serial sections for each heart. Error bars  =  s.e.m., significance tested by Student's t-test (41 dpi and 60 dpi) and Mann-Whitney rank sum test (others). n = 5 hearts 3 dpi (73 sections), 6 hearts 41dpi (89 sections), 7 hearts 60 dpi (118 sections). (F) Quantification of the ventricular area of the hearts analysed in E. Measurements were done on all sections for each heart. Significance tested by Student's t-test (41 dpi and 60 dpi) and Mann-Whitney rank sum test (others).

To establish a heart cryoinjury model, we exposed the apex of the ventricle by surgically opening the body wall and pericardial sac and froze the ventricle by application of dry ice. Fish mortality was <1%, which is lower than the mortality after ventricular resection in our hands. Furthermore, cryoinjured fish showed little impairment in swimming and feeding behaviour, indicating that the cryoinjury was well tolerated. Histological analysis of injured hearts stained with Acid Fuchsin Orange G (AFOG), which labels myocardium orange, collagen blue and fibrin red showed reduced tissue organization, loss of myocardium and massive accumulation of fibrin-rich wound tissue (red) four days post injury (dpi, Fig. 1B, insert 2) compared to control untreated hearts (Fig. 1A).

Importantly, cryoinjured fish were able to regenerate the damage. Histological analysis showed that by 14 dpi the size of the lesion had decreased and, importantly, there was no sign of collagen-rich scar tissue formation (Fig. 1C). Rather the lesion continued to appear enriched with fibrin (Fig. 1C, insert 2). By 60 dpi, the lesions had further dramatically decreased in size (Fig. 1D). We estimated the extent of recovery by measuring the size of the lesioned area in the histological section displaying the biggest wound in each cryoinjured heart. Using this method, we found that at 4 dpi, the largest parts of the lesions reproducibly affected 25% of the ventricle (Fig. S1A). Thus, zebrafish tolerate large heart lesions, similar to cryoinjured mice [19]. At 14 dpi, the upper injury limit had dropped to 10%, and at 60 dpi to 2% (Fig. S1A).

To quantify the lesions and the regeneration process more precisely, we repeated the whole experiment, performed serial sections of entire hearts at 3 dpi, 41 dpi and 60 dpi, and measured the lesioned area and the whole ventricle area on all sections. The largest parts of the lesions affected 26% of the ventricle area (data not shown), which is very similar to the upper lesion limit seen in the first experiment, indicating that our cryoinjury procedure results in highly reproducible damage. Calculation of the lesioned area of entire hearts revealed that on average 15% of the ventricle were affected at 3 dpi (Fig. 1E). The lesion had dramatically reduced in size to 3% of the ventricle at 41 dpi and to 0.5% at 60 dpi (Fig. 1E). Thus, the lesion size dropped 30-fold within 60 days. The average size of the ventricle stayed the same in both sets of experiments, indicating that damaged tissue had been replaced (Fig. 1F and Fig. S1B).

At 60 dpi, the small remaining lesioned tissue was interspersed with myocardial cells (arrowheads in Fig 1D, insert 2), indicating that newly formed cardiomyocytes had penetrated the lesion. While 3 out of 12 analyzed fish showed complete regeneration without any sign of collagen deposition at 60 dpi, small patches of collagen could be detected in 75% of the analyzed hearts, indicating that some scar tissue had formed (Fig. 1D, insert 2). This is in agreement with the regenerative success after ventricular resection, where small collagen deposits also have been reported at 60 days post resection [4]. The speed of regeneration after resection and cryoinjury appears to be similar as well, since after resection of 20% of the ventricle, 14% of the ventricle are still missing at 30 days post resection as reported by Poss et al. [4], while we find that the upper lesion size after cryoinjury of 26% of the ventricle was reduced to 12% at 41 days post injury. However, the blood clot/wound tissue formed after ventricular resection appears to be removed faster than the necrotic lesions caused by cryoinjury, since we found that at 30 days post resection of 20% of the ventricle, the upper size limit of the remaining detectable wound tissue was 2% (data not shown), and 12% after cryoinjury at 41 dpi. We conclude that zebrafish can regenerate cryoinjury-induced myocardial lesions with little scar formation.

### Cryoinjury causes necrotic cell death

**Figure 2:**
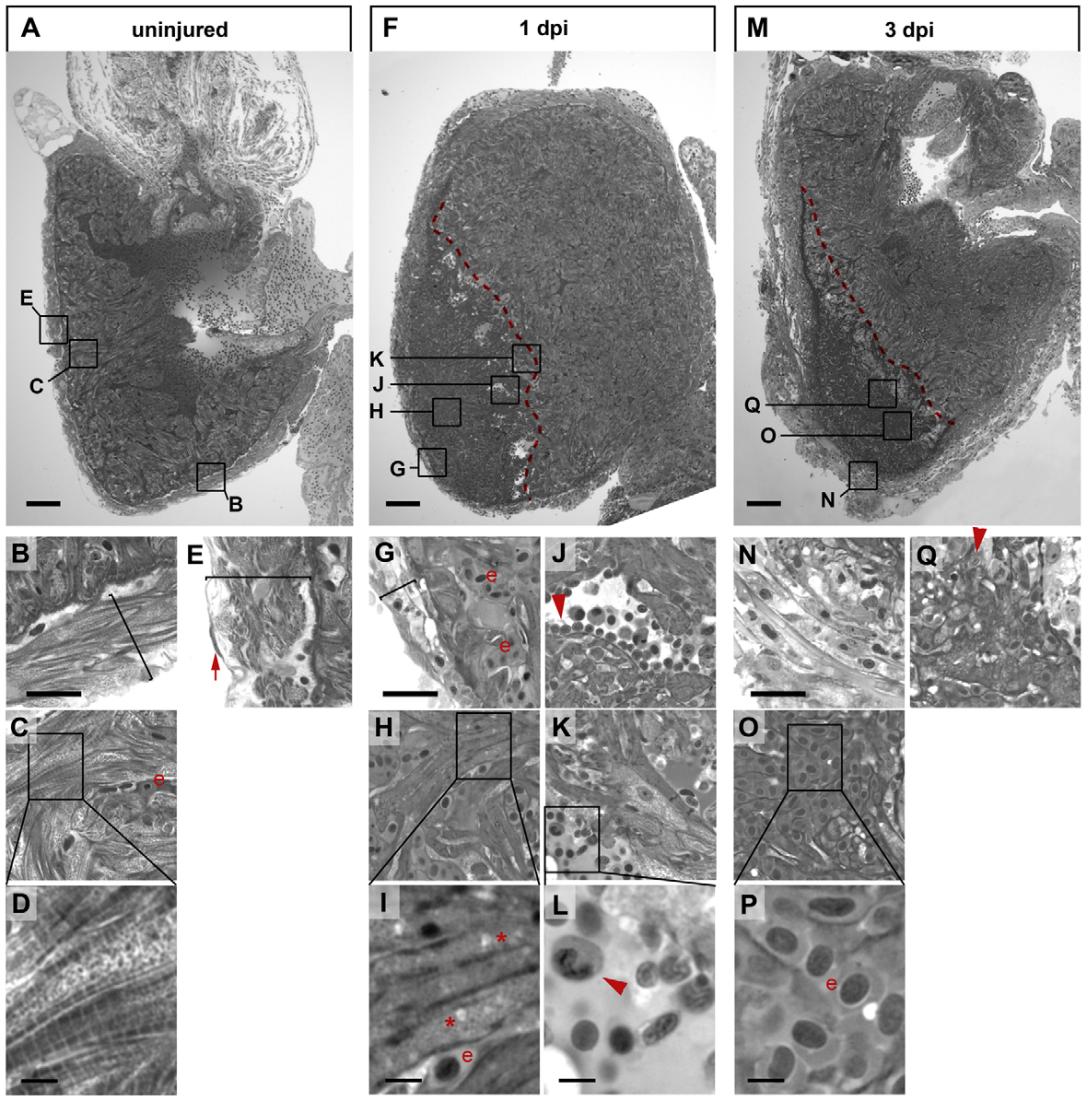
Cryoinjury causes cardiomyocyte death, inflammatory response and massive changes in tissue morphology. Semi-thin histological sections of an uninjured heart (A–E) and cryolesioned hearts at 1 dpi (F–L) and 3 dpi (M–Q) stained with Tuloidin blue are shown. Wound edges are indicated with red dashed lines (F, M). Note striated cardiomyocytes in the compact external (B) and in the trabeculated internal (C, D) myocardium in the uninjured heart and the single layer of epicardial epithelium covering the myocardium (red arrow in E). (F–L) At 1 dpi, the thickness of the external myocardial layer is reduced (compare brackets in B and G) in the lesioned area, and most cardiomyocytes have lost striations suggesting myofibril disassembly (G, H, I). Also note appearance of large vacuolar structures (asterisk in I) indicative of cell death. Leukocytes, largely heterophil granulocytes, delineate the wound edges (J, K, L; see red arrowheads). (M–Q) At 3 dpi the external ventricular layer displays a loosened morphology but is devoid of cardiomyocytes (N). At 3 dpi the lesioned area is largely filled with erythrocytes (“e” in O, P). Cellular debris in the wound area is often associated with granulocytes (red arrowhead in Q). For each time point n = 3 hearts. Scale bars in A, F and M are 100 µm. Scale bars in B, G and N are 25 µm. Scale bars in D, I, L and P are 5 µm.

Acridine orange staining of live cryoinjured hearts at 1 dpi showed intense staining at the injured ventricular apex, indicative of cell death (Fig. S2). To characterize the cellular damage caused by cryoinjury in more detail, we analyzed semi-thin plastic sections stained with toluidine blue. In uninjured hearts, the exterior of the ventricle was composed of a compact layer of cardiomyocytes (brackets in Fig. 2B and 2E), which was clearly demarcated from internal cardiomyocytes that were organized into trabeculae (Fig. 2A–C). Cardiomyocytes showed characteristic striations or a granular structure depending on their orientation relative to the plane of section (Fig. 2C, D) and they contained nuclei with a prominent nucleolus (data not shown). The epicardial epithelium surrounding the myocardium was evident as a single cell layer (arrow in Fig. 2E). The intra-trabecular space was filled with erythrocytes (e in Fig. 2C). One day after cryoinjury, the external myocardial layer was reduced in width and devoid of cells displaying cardiomyocyte morphology (Fig. 2F, bracket in Fig. 2G). Likewise, most myocardial cells in the affected trabecular area had lost their typical striated morphology (Fig. 2H, I) and characteristic nuclei, and displayed vacuolar structures indicative of cell death (asterisks in Fig. 2I) [20]. Furthermore, erythrocytes were strongly enriched in the lesioned area (e in Fig. 2G, I). The wounded area was found to be infiltrated with leukocytes, most of which displayed the characteristic nuclear morphology of neutrophil granulocytes (arrowheads in Fig. 2J, L). This indicates an induction of an inflammatory response to clear cellular debris from the affected area. During the following days the wound area was further remodeled. At 3 dpi the morphology of the ventricular surface had changed to a thickened layer with a loose appearance due to prominent intracellular space and the absence of tightly packed cardiomyocytes (Fig. 2M, N). The inner part of the lesion was predominated by erythrocytes (Fig 2O and e in Fig. 2P). No cardiomyocytes were found in the lesioned area, rather cell debris (Fig. 2Q) that was often closely associated with granulocytes (arrowhead in Fig. 2Q).

**Figure 3:**
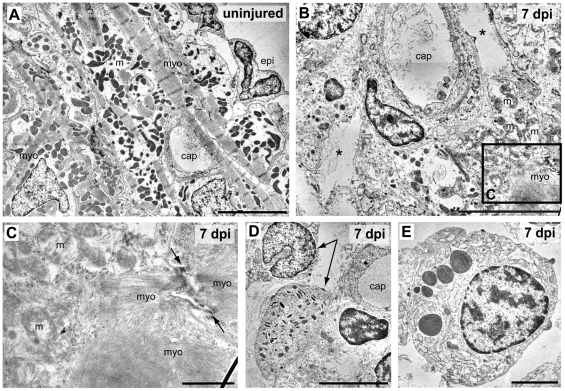
Ultrastructural analysis of cryolesioned myocardium reveals cardiomyocyte cell death and an inflammatory response. Electron micrographs of uninjured (A) and cryolesioned (7 dpi, B–E) zebrafish heart tissue. A normal organization of ventricular periphery with epicardal epithelium (epi) and subepicardial cardiomyocytes displaying well-organized striated myofilaments (myo) with Z lines and groups of electron dense mitochondria (m). A capillary (cap) is visible as well. (B) At 7 dpi, the lesioned area displays cellular debris and large tissue gaps (*) around a small capillary. In the lower right corner: cardiomyocyte with damaged mitochondria (m) and disorganised myofilaments (myo). (C) Magnification of the boxed area in B. A cryoinjured cardiomyocyte with disorganised mitochondria (m), myofilaments, and a loosened/ill defined intercalated disc (arrows) is shown. (D, E) Granulocytes in the wound area. (D) Heterophil granulocytes (arrows), the upper one with a peripheral, nonsegmented nucleus, the lower one with characteristic, cigar-shaped cytoplasmic granules. (E) Eosinophil granulocyte. Scale bars: 5 µm in A, B, and D, 1 µm in C, and 2 µm in E.

Electron microscopy of peripheral ventricular cell layers in uninjured hearts revealed subepicardial cardiomyocytes with prominent myofilaments and groups of electron dense mitochondria and a single layer of epicardial cells (Fig. 3A). In contrast, in the lesioned area of cryoinjured hearts at 7 dpi, cellular debris (Fig. 3B) and large tissue gaps (asterisk in Fig. 3B) could be detected. Furthermore, the lesion contained remnants of cardiomyocytes displaying highly disorganized myofilaments and damaged mitochondria (Fig. 3B, C). Electron micrographs also confirmed the presence of heterophil/neutrophil (arrows in Fig. 3D) and eosinophil granulocytes (Fig. 3E) in the lesion.

Overall, our histological and ultrastructural analyses indicate that cryoinjury resulted in necrotic cell death and loss of cardiomyocytes in the lesioned area, which was accompanied by infiltration of erythrocytes and leukocytes.

### All three heart layers are susceptible to cryoinjury

**Figure 4:**
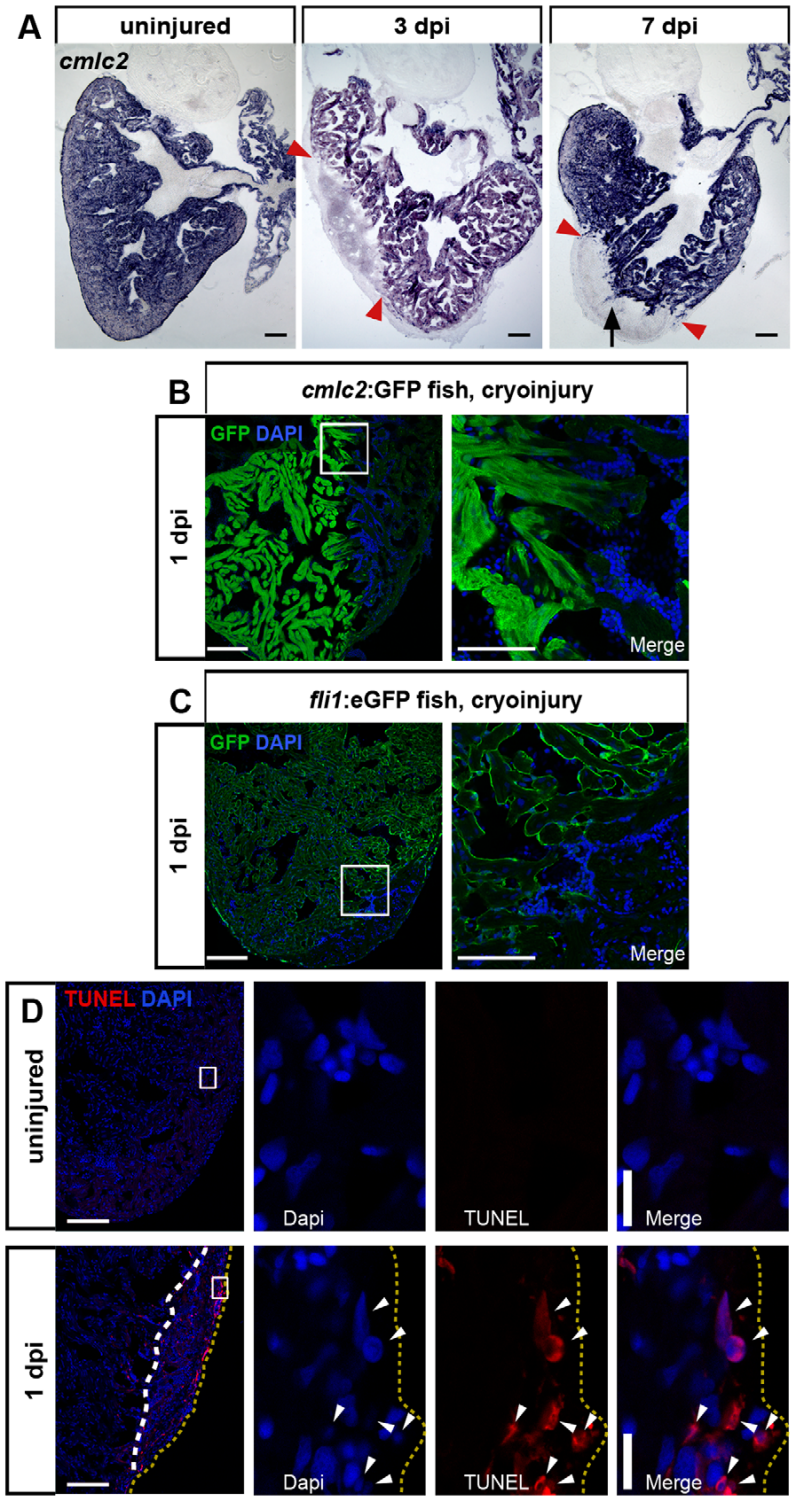
Cryolesion results in loss of cardiomyocyte and endocardium-specific marker gene expression and in induction of apoptosis. (A) In situ hybridization shows loss of *cmlc2* (*myl7*) expression in the lesioned area at 3 and 7 dpi. The lesioned area is indicated by red arrowheads. Note the protrusions of *cmlc2* positive areas (black arrow) extending into the lesion at 7 dpi. Scale bars are 100 µm. n = 3 hearts (9 sections each) for each uninjured, 3 dpi and 7 dpi. (B) Loss of *cmlc2* expression is already evident in cryoinjured hearts at 1 dpi of *cmlc2*:GFP transgenic fish stained for GFP by immunofluorescence. Nuclei are stained with Dapi. Scale bar in overview is 100 µm, 25 µm in close-up. (C) Endocardial GFP expression is lost in cryolesions of *fli1*:eGFP transgenic fish at 1 dpi. (D) TUNEL assay on hearts at 1 dpi detects an increased number of apoptotic cells (white arrowheads) compared to uninjured hearts. Wound area is indicated by white dashed line and the periphery of the heart is marked by yellow dashed lines. Nuclei are stained with Dapi. Scale bar in overview is 100 µm. Scale bar in close up is 10 µm. n = 3 hearts each uninjured and 1 dpi.

Loss of cardiomyocytes in the cryoinjured area was further evident by loss of transcripts for the cardiomyocyte specific gene *cardiac myosin light chain 2 (cmlc2, myl7)* (Fig. 4A). At 3 dpi, the lesioned area was completely devoid of *cmlc2* positive cells (Fig. 4A). Expression had not recovered at 7 dpi. However, while the *cmlc2*-negative region was clearly demarcated from the *cmlc2*-expressing myocardium at 3 dpi, at 7 dpi scattered *cmlc2*-positive cells appeared at the injury border zone and protrusions of *cmlc2*-positive areas were found to extend into the lesion (arrow in Fig. 4A). These data indicate that by 7 dpi cardiomyocytes had started to invade the lesioned area. Damage to *cmlc2*-expressing CMs was already evident at 1 dpi as seen by loss of GFP fluorescence in *cmlc2*:GFP transgenic fish (Fig. 4B). We thus asked whether the endocardium is similarily affected. Indeed we found that GFP expression in *fli1*:eGFP transgenic fish [15], [21], which specifically labels the vascular endothelium and endocardium, was lost in the lesioned area at 1 dpi (Fig. 4C).

We asked whether apoptosis contributes to cell death after cryoinjury as well. While we could only very rarely detect apoptotic cells in uninjured hearts, a sizable number of cells was TUNEL-positive in the lesioned area at 1 dpi (Fig. 4D). Thus, both necrosis and apoptosis lead to myocardial tissue loss after cryoinjury. Interestingly, some of the TUNEL-positive cells were located on the outer surface of the heart (yellow dashed lines), suggesting their epicardial identity (boxed area, Fig. 4D).

In summary, these data suggest that epicardium, myocardium and endocardium are susceptible to apoptotic and necrotic cell death in response to cryoinjury.

### Organ-wide response of the epicardium to cryoinjury

**Figure 5:**
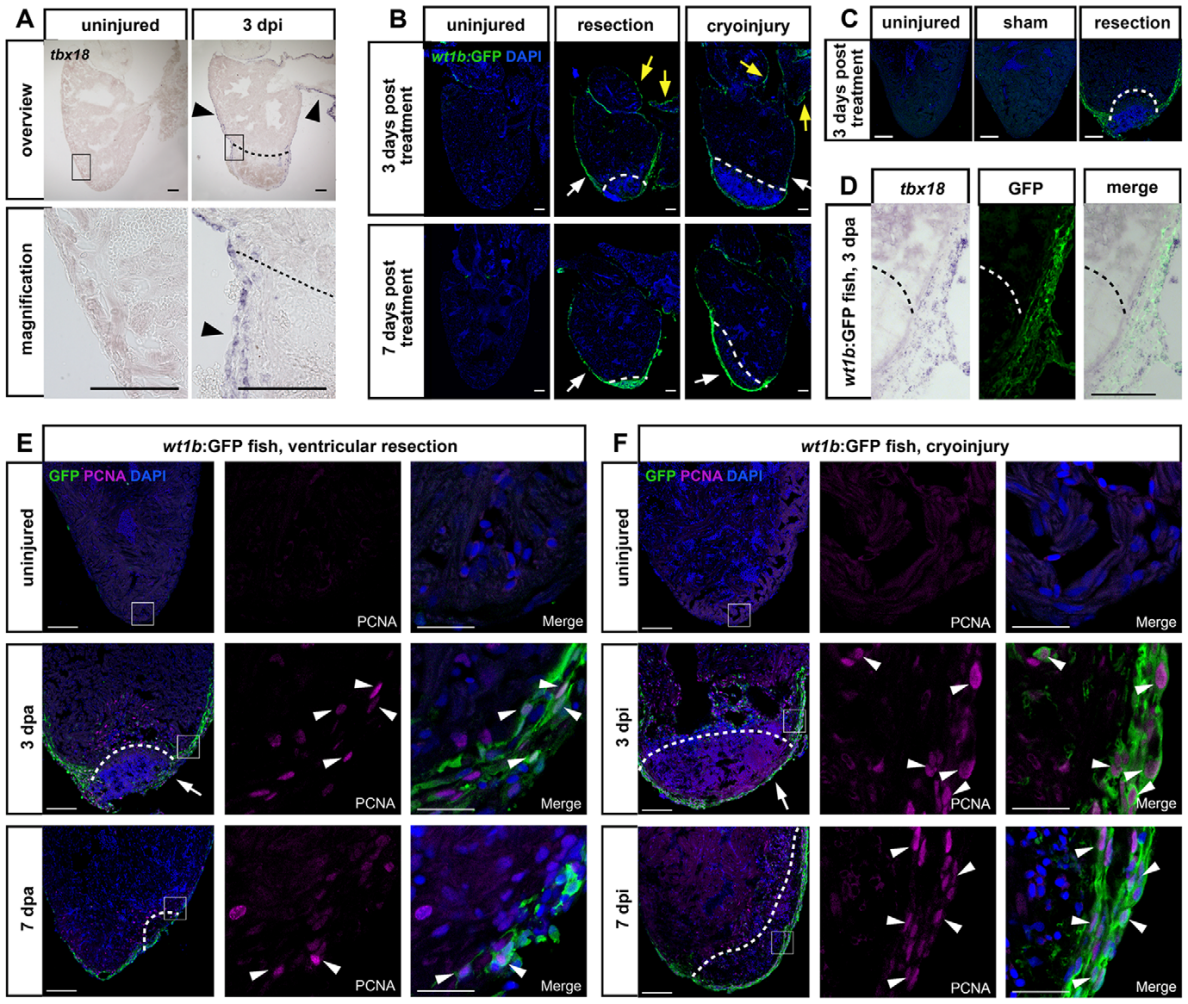
Organ-wide activation of embryonic gene expression and cell proliferation in the epicardium in response to cryoinjury and ventricular resection. (A) In situ hybridization for *tbx18* shows an organ-wide expression (arrowheads) in the epicardium 3 days after cryoinjury compared to undetectable expression in uninjured hearts. Wound edges are indicated by black dashed lines. Scale bars are 100 µm. n = 3 heart control and 3 dpi. (B) Organ-wide GFP induction in the transgenic *wt1b*:GFP line shows activated epicardium after ventricular resection and after cryoinjury at 3 and 7 days after treatment. Induction of the transgene is visible in the thickened epicardium near the wound (white arrows) and in the epicardium lining the bulbus arteriosus and atrium as well (yellow arrows). Uninjured hearts show no GFP expression. Nuclei were stained with Dapi. Wound edges are indicated by white dashed lines. Scale bars are 100 µm. n = 5 hearts at all time points. (C) Organ-wide GFP induction in the transgenic *wt1b*:GFP line is only observed after ventricular resection but not in uninjured or sham amputated control hearts. Nuclei were stained with Dapi. Scale bars are 100 µm. (D) GFP expression in injured *wt1b*:GFP transgenic hearts is specific to the epicardium. Coexpression of *tbx18* transcript detected via in situ hybridization and GFP detected via antibody staining at 3 days post amputation is shown. Scale bar is 100 µm. n = 3 hearts. Dashed line indicates wound edge. (E–F) Activation of epicardial proliferation in response to ventricular resection (E) and cryoinjury (F). Uninjured and amputated hearts at 3 dpa and 7 dpa (E) or cryoinjured hearts at 3 dpi and 7 dpi (F) of *wt1b*:GFP fish were stained for GFP and PCNA expression. Nuclei are stained with Dapi. Proliferating epicardial cells are indicated with white arrowheads. Note partial coverage of the wound with GFP-positive epicardium in E (arrow) and full coverage in F (arrow). Scale bars are 100 µm in the overview and 25 µm in the close ups. Wound plane is indicated by white dashed lines. n  = 3 hearts (9 sections) for all conditions.

One early response to zebrafish ventricular resection is an activation of the epicardium, which occurs in the entire heart and results in upregulation of a developmental gene program and induced cell proliferation [15]. We thus asked whether the epicardium responds similarly to ventricular cryoinjury. The *tbx18* transcription factor is expressed in the epicardium during zebrafish embryonic development [22], but is not detectable in the uninjured adult epicardium (Fig. 5A). In contrast, cryoinjury activated *tbx18* transcription in the entire epicardium by 3 dpi (arrowheads in Fig. 5A). The *wilms tumor 1* (*wt1*) gene is another marker for the developing vertebrate epicardium [23]; in zebrafish, both *wt1* paralogs (*wt1a* and *wt1b*) have been reported to be expressed in the adult heart [24], [25]. We thus asked whether a zebrafish transgenic line expressing GFP under control of *wt1b* regulatory sequences [24] reports activation of the epicardium after injury. We first tested whether the transgene is activated in response to ventricular resection (“amputation”). While no GFP activity could be detected in uninjured hearts, strong GFP expression was evident in the outermost cell layers of the heart at 3 days post amputation (dpa) and still detected at 7 dpa (white arrows in Fig. 5B). Likewise, GFP activity was found in cryoinjured hearts at these stages (white arrows in Fig. 5B). After both types of injuries organ-wide upregulation of *wt1b*:GFP was seen that included the entire surface of the ventricle, and also the atrium and the bulbus arteriosus (yellow arrows in Fig. 5B). Similar to uninjured controls, sham amputated hearts, in which the pericardial sac had been opened but the ventricle left untouched, displayed no GFP activity, showing that *wt1b* upregulation was injury specific and not a general stress response (Fig. 5C). To confirm that GFP expression in this transgenic line is confined to epicardial cells, we co-labelled sections of amputated hearts for *tbx18* RNA and GFP protein at 3 dpa. We found that expression overlapped (Fig. 5D), indicating that the *wt1b*:GFP transgenic line is a useful tool to label injury-activated epicardial cells in the adult zebrafish heart.

In amputated hearts of *wt1b*:GFP transgenic fish, a thickening of the GFP+ cell layer was evident at the wound edges, and the blood clot sealing the wound was partially overlain by GFP+ cells at 3 dpa (arrow in Fig. 5E), suggesting that epicardial cells had moved into and covered part of the wound. Many GFP+ cells were proliferating as indicated by co-labelling with PCNA (Fig. 5E). At 7 dpa, GFP positive cells that partly co-labelled with PCNA were found covering the entire wound area, suggesting that by that stage the epicardial outer surface of the heart had been restored (Fig. 5E). Interestingly, in cryoinjured *wt1b*:GFP transgenic hearts, GFP positive cells were detected at the outer surface of the entire injured area already by 3 days post injury (arrow in Fig. 5F), suggesting that epicardial cells migrated and covered the cryoinjury-induced lesion more quickly than after ventricular resection. Proliferation was also induced in epicardial cells after cryoinjury, as evident by a large number of *wt1b*:GFP positive cells that expressed PCNA (Fig. 5F). GFP activity was sustained at 7 dpi, and epicardial cells likewise remained proliferative (Fig. 5F).

Together, these data suggest that cryoinjury results in an organ-wide activation of a developmental gene program and induction of cell proliferation in the epicardium. Thus, cryoinjury elicits a similar response as ventricular resection. The timing and amplitude of epicardial gene expression and proliferation activation appear to be comparable between both injury models, yet epicardial cells seem to be able to cover the lesion more quickly after cryoinjury.

### Induction of cardiomyocyte proliferation

**Figure 6:**
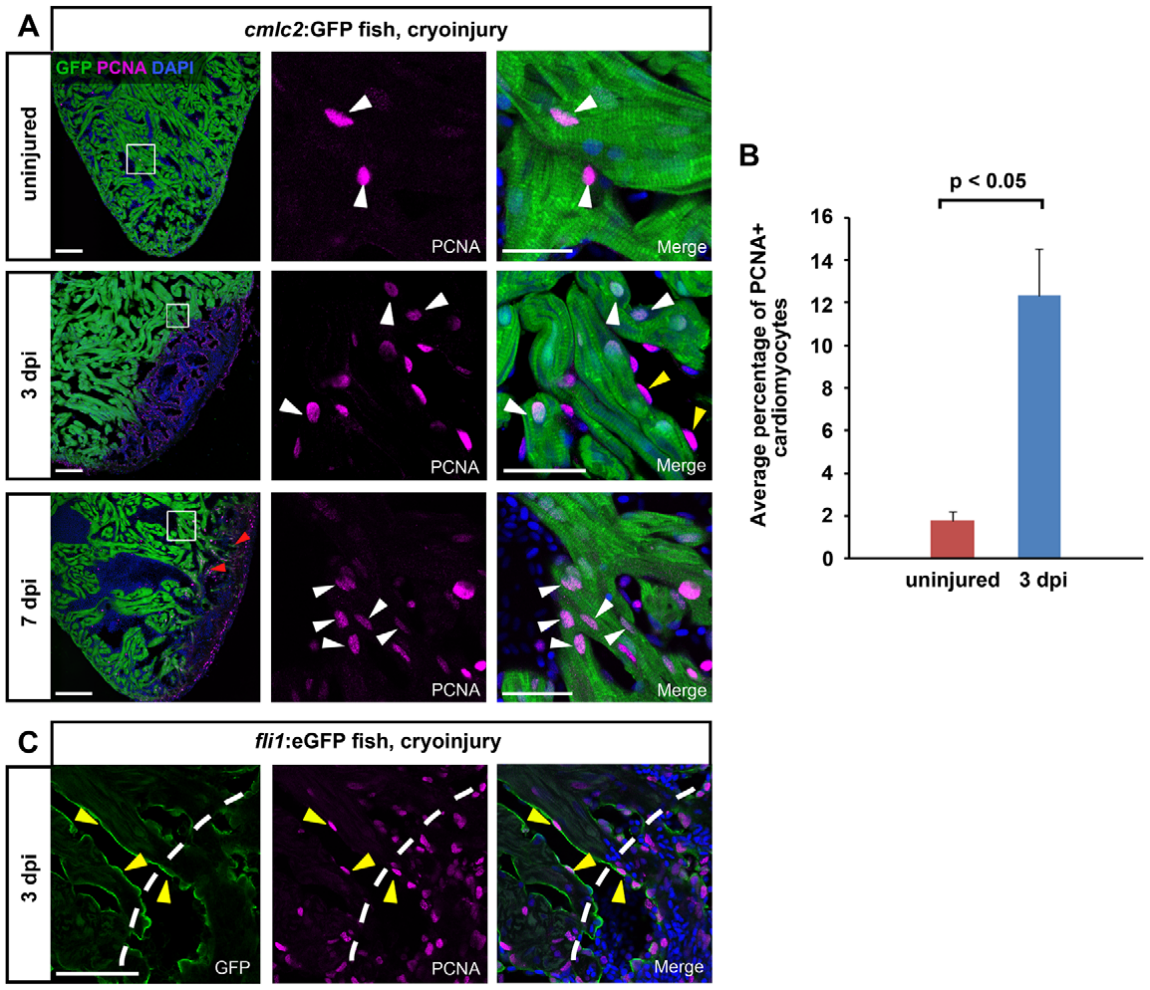
Activation of cardiomyocyte proliferation in response to cryoinjury. (A) Mature cardiomyocytes located close to the lesion in the uninjured myocardium proliferate at 3 and 7 dpi. Uninjured and cryolesioned hearts (3 dpi and 7 dpi) of *cmlc2*:GFP transgenic fish were stained for GFP and PCNA. Nuclei are stained with Dapi. Proliferating cardiomyocytes are indicated by white arrowheads. Proliferating endocardial cells are indicated by yellow arrowheads (3 dpi). Note individual GFP+ cardiomyocytes inside the lesioned area at 7 dpi (red arrowheads). Scale bars are 100 µm in the overview and 25 µm in the close ups. n = 3 hearts (9 sections) at all conditions. (B) Quantification of the percentage of PCNA+ cardiomyocytes in uninjured versus cryolesioned hearts at 3 dpi. CMs were quantified in the entire ventricle. Error bars  =  s.e.m. n = 3 hearts (7 sections for 3 dpi, 5260 CMs counted; 9 sections for uninjured control, 7475 CMs counted), Student's t-test was employed for statistical analysis, with p<0.05. (C) Endocardial cells located adjacent to the lesion proliferate at 3 dpi. Cryolesioned hearts (3 dpi) of *fli1*:eGFP transgenic fish were stained for GFP and PCNA. Nuclei are stained with Dapi. Proliferative endocardial cells are indicated by yellow arrowheads. Wound edge is indicated by a white dashed line. Scale bars are 25 µm.

In response to ventricular resection, zebrafish cardiomyocytes have been shown to become proliferative and recent lineage tracing data indicate that the majority of the regenerating myocardium is derived from mature cardiomyocytes [15], [16], [17]. To test whether cryoinjury likewise results in cardiomyocyte proliferation, we stained *cmlc2*:GFP transgenic hearts for PCNA. Less than 2% of the cardiomyocytes were PCNA positive in uninjured adult hearts (Fig. 6A, B). At 1 and 3 dpi, the lesioned area was devoid of GFP due to the aforementioned loss of CMs (Fig. S3 and Fig. 6A). At 1 dpi, no increase in the number of PCNA positive cardiomyocytes could be detected in the noninjured areas of the ventricle (Fig. S3). In contrast, at 3 dpi, a large number of cells in the injured heart were positive for PCNA, including *cmlc2*:GFP positive cardiomyocytes found close to the lesion in the uninjured myocardium (white arrowheads in Fig. 6A). Cardiomyocyte proliferation had increased 6 fold at 3 dpi relative to injured hearts, with 12% of all ventricular CMs expressing PCNA (Fig. 6B). Thus, cryoinjury induced an early proliferative response of differentiated cardiomyocytes in the non-lesioned myocardium. In addition, nuclei of GFP-negative cells found in close association with cardiomyocytes in the uninjured areas were also expressing PCNA, indicating that endocardial cells likewise became proliferative (yellow arrowheads in Fig. 6A). To confirm this, we stained cryoinjured hearts of *fli1*:eGFP transgenic fish for PCNA. Indeed we detected proliferating endocardial cells lining the trabeculated uninjured myocardium close to the lesioned area in cryoinjured hearts at 3 dpi (Fig. 6C). Thus, all three layers of the heart start to proliferate in response to cryoinjury.

At 7 dpi, individual GFP-positive cardiomyocytes and extensions of GFP+ myocardium were found in the lesioned area (red arrowheads in Fig. 6A), supporting the above mentioned conclusion that cardiomyocytes had started to invade the injured tissue by 7 dpi. Moreover, a significant number of cardiomyocytes was positive for PCNA at this stage (Fig. 6A). Thus, cryoinjury of the zebrafish heart resulted in induction of myocardial proliferation and invasion of the lesion with proliferative cardiomyocytes.

### Conclusions

We describe a simple method for induction of necrotic lesions in the adult zebrafish heart based on cryoinjury. Despite widespread tissue death and loss of cardiomyocytes, epicardial and endocardial cells caused by these lesions, zebrafish display a robust regenerative response, which results in substantial clearing of the necrotic tissue and little scar formation. The cellular mechanisms underlying this regenerative response appear to be similar to the ones utilized during regeneration of ventricular resections. Early after injury, the entire epicardium activates a developmental gene program and becomes proliferative. We have found that epicardium activation in response to ventricular resection and cryoinjury is robustly reported by a *wt1b*:GFP transgenic zebrafish line, which thus represents a useful tool for future studies of heart regeneration. After both types of injury, the activated epicardial cells cover the lesioned area, presumably by migration. We found that the latter process is completed earlier in cryoinjured hearts than after ventricular resection. Whether this is due to an intrinsic differential response of epicardial cells induced by the type of injury or based on the properties of the lesioned area, eg. a consequence of the properties of the cellular and acellular substrates that the epicardial cells have to migrate on, remains to be tested. We did not detect significant differences in the timing or amplitude of gene expression induction or upregulation of proliferation in epicardial cells in response to the two types of injury, indicating that intrinsic differences in the epicardial response are less likely to be causative for the observed difference in wound coverage. However, after ventricular resection, the wound tissue adheres more strongly to the pericardial sac than after cryoinjury. It is possible that wound coverage by the epicardium is impaired by this adherence.

Regeneration of myocardium removed by ventricular resection appears to occur via proliferation of differentiated cardiomyocytes [15], [16]. We likewise find that mature cardiomyocytes, expressing *cardiac myosin light chain (cmlc2, myl7)* proliferate in response to cryoinjury and that proliferating cardiomyocytes invade the lesioned area. These data strongly indicate that necrotic lesions are repaired by proliferation of existing mature cardiomyocytes.

Overall, our work shows that zebrafish cannot only restore surgically removed heart tissue, but also regenerate necrotic lesions. Since the latter type of injury is closer to the cardiac tissue damage seen in human patients, our results underscore the relevance of research into the cellular and molecular mechanisms of natural heart regeneration in the zebrafish for efforts to devise regenerative therapies in humans. While we find that both ventricular resection and cryoinjury induced lesions are repaired using similar cellular mechanisms, we noticed a few temporal differences. Combined with the fact that we find setting of cryoinjuries to be less demanding of the experimenter and better tolerated by the fish than ventricular resection, we expect that this injury model will be highly valuable for future research into the molecular mechanisms of zebrafish heart regeneration.

## Methods

All animal experiments have been performed in accordance with the guidelines of the state of Saxony and have been approved by the Regierungspräsidium Dresden (permit number 24-9168.11-1/2008-1).

### Transgenic fish lines

To visualize cardiomyocytes, we used transgenic zebrafish expressing GFP under control of the *cmlc2* (*myl7*) promoter (*cmlc2*:GFP, [26]), to label activated epicardial cells we used *wt1b*:EGFP^li1^ transgenic fish [24] and to detect endocardial cells we used *fli1*:eGFP^y1^ transgenic fish [21].

### Ventricular resection

During ventricular resections ∼20% of ventricular tissue was removed from the apex using iridectomy scissors as described previously [4].

### Cryoinjury

Fish were anesthetized with 0.02% Tricaine (MS-222) and transferred to a moist sponge for surgery. After visually locating the posterior medial margin of the heart straight iridectomy scissors were used to puncture the skin and the silvery pericardial sac. Subsequently, an incision was made through both the skin and pericardium starting from the junction of the pericardium and peritoneum and reaching anteriorly for about 2/3 of the length of the heart. The incision was spread open laterally using fine forceps to expose the ventricle. Small pieces of dry ice were formed into a conical shape with a length of ∼20 mm, one end with a diameter of ∼2 mm and the other end with a pointed tip. The pointed tip of the dry ice cone was applied to the posterior apex of the ventricle for 10 seconds to cause the cryoinjury. After surgery the fish were returned to holding tanks. To revitalize the fish a pipette was used to vigorously squirt water over the gills until the fish started to breathe regularly. Sham treated control fish in which the pericardial sac was opened but the heart left untouched showed no signs of necrosis, induction of *wt1b*:GFP expression or upregulation of cardiomyocyte proliferation. Thus, hearts of untreated fish served as uninjured control samples in most experiments.

### Histological methods and electron microscopy

Acid fuchsin-orange G (AFOG) staining was performed on cryosections as described [4]. Measurements of wound area and ventricle area on cryosections were performed with the Fiji distribution of the ImageJ (NIH) software.

For semi-thin sections and electron microscopy, zebrafish hearts were fixed in modified Karnovsky's fixative (2% glutaraldehyde +2% paraformaldehyde in 50 mM HEPES, [27], [28]) at 4°C overnight, and washed 2x in 100 mM HEPES and 2x in PBS. For light microscopy the samples were embedded in the methacrylate Technovit 7100 (Heraeus-Kulzer). They were transferred to a microwave-assisted tissue processor (Leica-EM AMW) and processed according to the following protocol: PBS (2x 3 min at 35°C, 15 W), 1% OsO_4_ in PBS (30 min at 50°C, 25 W, pulse: 10 s MW on, 50 s MW off), PBS (4 min at 37°C, 15 W), 2x water (4 min at 37°C, 17 W), 30%, 50%, 70%, 95% and 2x 100% ethanol (5 min each at 37°C, 15 W/11 W), Technovit 7100:ethanol (1∶1) (15 min at 37°C, 11 W), Technovit 7100:ethanol (2∶1, 3∶1) (20 min each at 37°C, 11 W), pure resin (20 min at 37°C, 11 W). After the AMW run, samples were transferred to fresh Technovit resin overnight, and finally embedded. 2 µm sections were mounted on glass slides and stained with 1% toluidin blue 0.5% borax. For electron microscopy, the specimens were postfixed with 1% OsO_4_/water for 2 h on ice, washed with PBS and water, and *en bloc* contrasted with 1% uranyl acetate in water. The samples were then washed several times in water, dehydrated in a graded series of ethanol, infiltrated in epon 812 (epon/ethanol mixtures: 1∶3, 1∶1, 2∶1, 3∶1 1.5 h each, pure epon overnight, pure epon 3 h), and embedded in flat embedding molds. Ultrathin sections were collected on formvar-coated slot grids, stained with lead citrate and uranyl acetate according to Venable and Coggeshall [29], and analyzed on a FEI Morgagni 268 at 80 kV.

### *In situ* hybridization

ISH on cryosections of hearts fixed in 4% paraformaldehyde was performed using digoxygenin-labeled RNA probes of *tbx18* and *cmlc2* (*myl7*) as described [4]. For costaining with anti GFP antibody, sections were initially stained for *tbx18* expression using in situ hybridization. This was followed by several washes in PBT (1xPBS, 0.2% Tween-20) and blocking in NCS-PBT (10% newborn calf serum, 1% DMSO, PBT). Incubation of anti-GFP antibody (1∶1500; Abcam #ab13970) was performed over night at 4°C. After several washes in PBT secondary anti-Chicken Alexa 488 (1∶1000) was applied for 45 min. Following several washes in PBT, sections were mounted in 70% glycerol in PBS and analyzed with an Olympus upright microscope.

### Immunofluorescence

Hearts were extracted, fixed in 4% PFA (in Phosphate buffer) and cryosectioned into 14 µm thin sections. PEMTx buffer (80 mM Na-PIPES, 5 mM EGTA, 1 mM MgCl_2_, pH 7.4; 0.2% Triton-100) was used for immunohistochemistry. Primary antibodies were anti-PCNA (1∶5000; Dako #M0879) and anti-GFP (1∶1500; abcam #ab13970). Secondary antibodies conjugated to Alexa 488 or 633 (Invitrogen) were used at a dilution of 1∶1000. Nuclei are shown by DAPI (4′,6-diamidino-2-phenylindole) staining. Confocal images were acquired using a Leica Sp5 confocal microscope.

### Detection of necrosis and apoptosis

For acridine orange staining, zebrafish hearts were incubated in acridine orange diluted in PBS (2 µg/ml, Invitrogen) for 1 h, washed twice in PBS for 10 min, and imaged with a MZ16 FA fluorescence stereomicroscope (Leica).

TUNEL staining was performed on 14 µm cryosections using the ApopTag Red *in situ* Apoptosis Detection Kit (Chemicon) according to the manufacturer's instructions.

## Supporting Information

Figure S1 **Quantification of the largest extent of cryolesions and the size of the ventricle.** (A) Quantification of the upper limit of the lesioned area size normalized to the size of the ventricle in experimental set 1. Measurements were performed on the section displaying the biggest wound for each heart. Error bars  =  s.e.m., significance tested by Student's t-test. n = 5 hearts 4 dpi, 5 hearts 14 dpi, 4 hearts 60 dpi. (B) Quantification of the ventricular area at 4, 14 and 60 dpi of the sections analysed in A. One-way Anova test was used to show that ventricular areas are not significantly different.
(TIF)

Figure S2 **Acridin orange staining indicates cell death (red signal, arrow) in cryoinjured heart at 1 dpi compared to uninjured control.**
(TIF)

Figure S3 **Mature cardiomyocytes located close to the lesion in the uninjured myocardium are not proliferative at 1 dpi.** Cryolesioned hearts (1 dpi) of *cmlc2*:GFP transgenic fish were stained for GFP and PCNA. Nuclei are stained with Dapi. Scale bars are 100 µm in the overview and 25 µm in the close ups.
(TIF)
