# Supplementary figures and images for: Regeneration of Cryoinjury Induced Necrotic Heart Lesions in Zebrafish Is Associated with Epicardial Activation and Cardiomyocyte Proliferation
Source: PLoS One. 2011 Apr 12;6(4):e18503. doi: 10.1371/journal.pone.0018503 (PMC3075262; doi:10.1371/journal.pone.0018503)

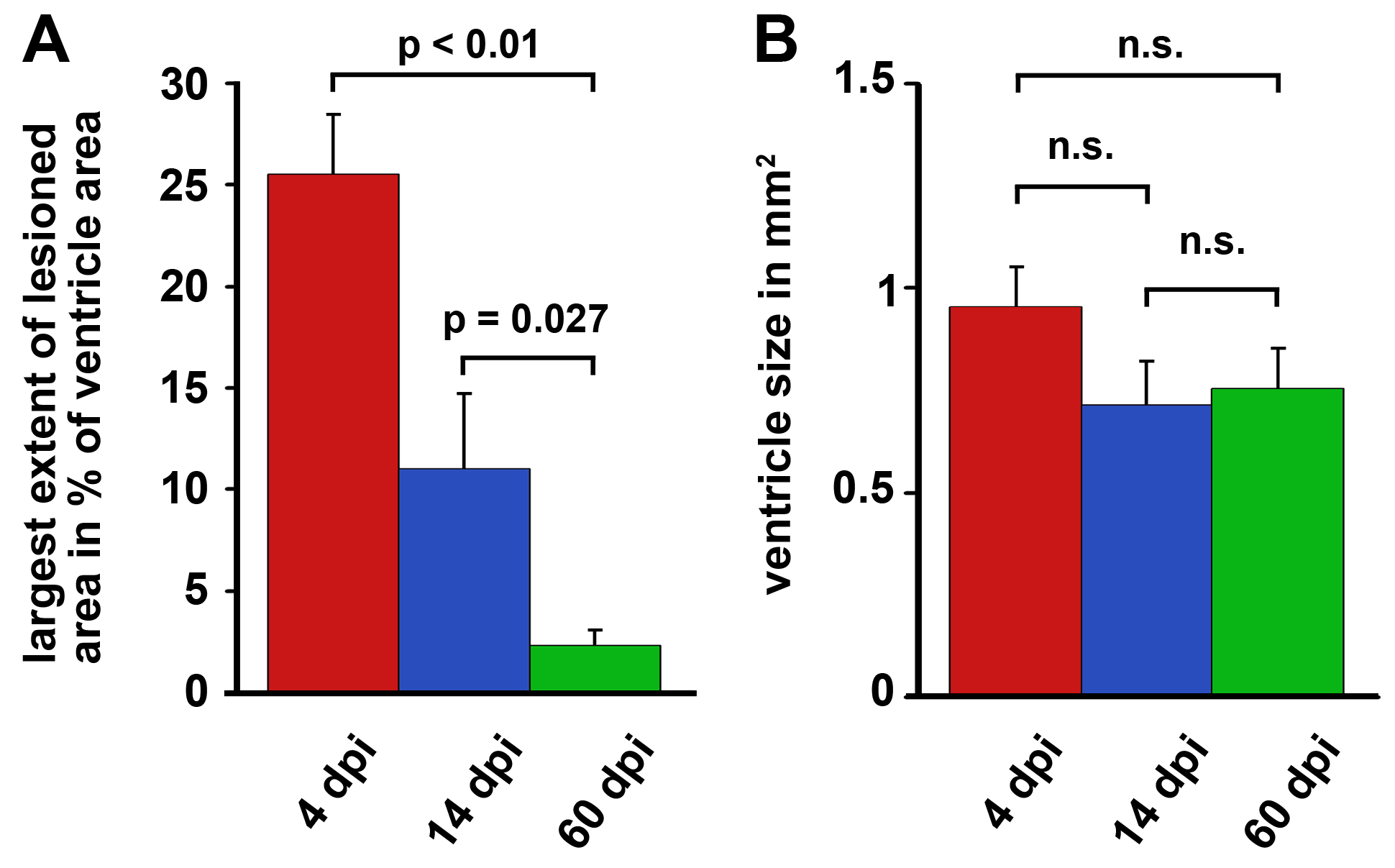

Supplement: Figure S1 — Quantification of the largest extent of cryolesions and the size of the ventricle. (A) Quantification of the upper limit of the lesioned area size normalized to the size of the ventricle in experimental set 1. Measurements were performed on the section displaying the biggest wound for each heart. Error bars = s.e.m., significance tested by Student's t-test. n = 5 hearts 4 dpi, 5 hearts 14 dpi, 4 hearts 60 dpi. (B) Quantification of the ventricular area at 4, 14 and 60 dpi of the sections analysed in A. One-way Anova test was used to show that ventricular areas are not significantly different. (TIF) [file pone.0018503.s001.tif]

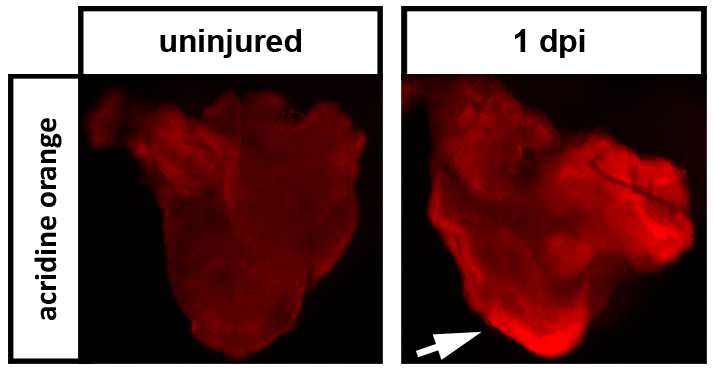

Supplement: Figure S2 — Acridin orange staining indicates cell death (red signal, arrow) in cryoinjured heart at 1 dpi compared to uninjured control. (TIF) [file pone.0018503.s002.tif]

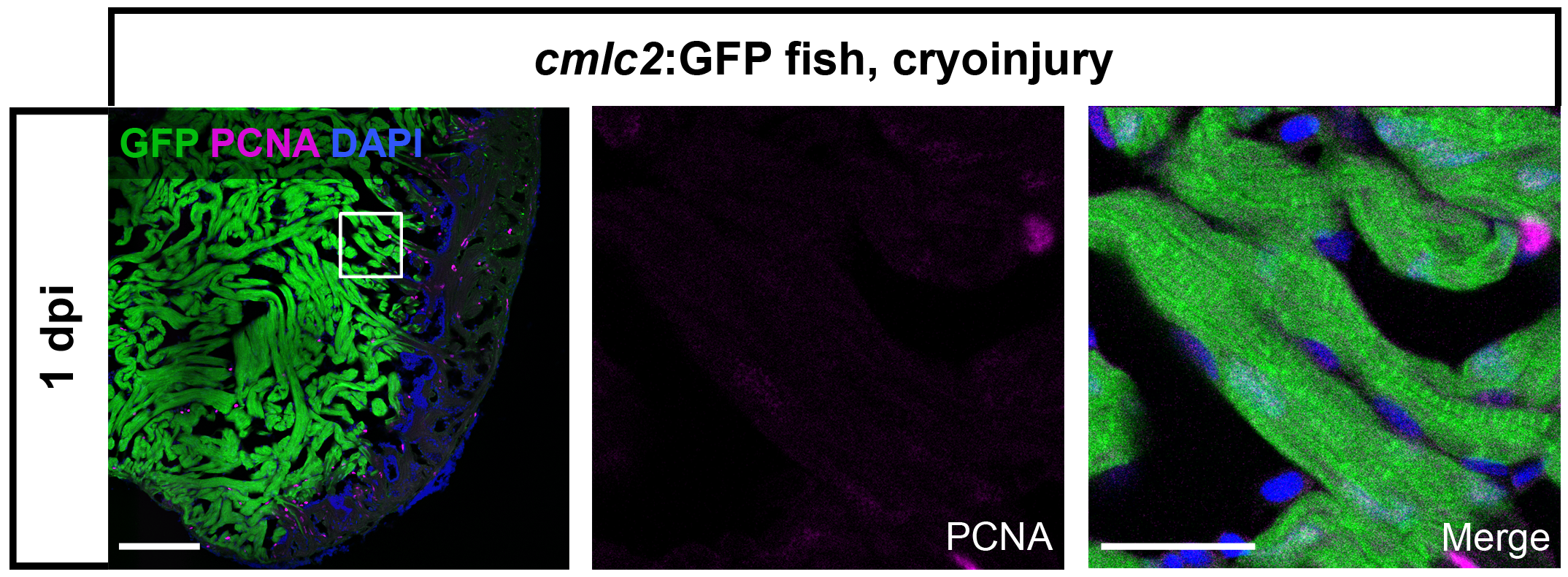

Supplement: Figure S3 — Mature cardiomyocytes located close to the lesion in the uninjured myocardium are not proliferative at 1 dpi. Cryolesioned hearts (1 dpi) of cmlc2:GFP transgenic fish were stained for GFP and PCNA. Nuclei are stained with Dapi. Scale bars are 100 µm in the overview and 25 µm in the close ups. (TIF) [file pone.0018503.s003.tif]
